# Neo-antigens predicted by tumor genome meta-analysis correlate with increased patient survival

**DOI:** 10.1101/gr.165985.113

**Published:** 2014-05

**Authors:** Scott D. Brown, Rene L. Warren, Ewan A. Gibb, Spencer D. Martin, John J. Spinelli, Brad H. Nelson, Robert A. Holt

**Affiliations:** 1 Canada’s Michael Smith Genome Sciences Centre, BC Cancer Agency, Vancouver, British Columbia V5Z 1L3, Canada;; 2 Genome Science and Technology Program, University of British Columbia, Vancouver, British Columbia V6T 1Z4, Canada;; 3 Department of Medical Genetics, University of British Columbia, Vancouver, British Columbia V6T 1Z4, Canada;; 4 Deeley Research Centre, BC Cancer Agency, Victoria, British Columbia V8R 6V5, Canada;; 5 Cancer Control Research Program, BC Cancer Agency, Vancouver, British Columbia V5Z 1L3, Canada;; 6 School of Population and Public Health, University of British Columbia, Vancouver, British Columbia V6T 1Z4, Canada;; 7 Department of Biochemistry and Microbiology, University of Victoria, Victoria, British Columbia V8P 5C2, Canada;; 8 Department of Molecular Biology and Biochemistry, Simon Fraser University, Burnaby, British Columbia V5A 1S6, Canada

## Abstract

Somatic missense mutations can initiate tumorogenesis and, conversely, anti-tumor cytotoxic T cell (CTL) responses. Tumor genome analysis has revealed extreme heterogeneity among tumor missense mutation profiles, but their relevance to tumor immunology and patient outcomes has awaited comprehensive evaluation. Here, for 515 patients from six tumor sites, we used RNA-seq data from The Cancer Genome Atlas to identify mutations that are predicted to be immunogenic in that they yielded mutational epitopes presented by the MHC proteins encoded by each patient’s autologous *HLA-A* alleles. Mutational epitopes were associated with increased patient survival. Moreover, the corresponding tumors had higher CTL content, inferred from *CD8A* gene expression, and elevated expression of the CTL exhaustion markers *PDCD1* and *CTLA4*. Mutational epitopes were very scarce in tumors without evidence of CTL infiltration. These findings suggest that the abundance of predicted immunogenic mutations may be useful for identifying patients likely to benefit from checkpoint blockade and related immunotherapies.

The accumulation of somatic mutations underlies the initiation and progression of most cancers by conferring upon tumor cells unrestricted proliferative capacity (Hanahan and Weinberg 2011). The analysis of cancer genomes has revealed that tumor mutational landscapes (Vogelstein et al. 2013) are extremely variable among patients, among different tumors from the same patient, and even among the different regions of a single tumor (Gerlinger et al. 2012). There is a need for personalized strategies for cancer therapy that are compatible with mutational heterogeneity, and in this regard, immune interventions that aim to initiate or enhance anti-tumor immune responses hold much promise. Therapeutic antibodies and chimeric antigen receptor (CAR) technologies have shown anti-cancer efficacy (Fox et al. 2011), but such antibody-based approaches are limited to cell surface target antigens (Slamon et al. 2001; Coiffier et al. 2002; Yang et al. 2003; Cunningham et al. 2004; Kalos et al. 2011). In contrast, most tumor mutations are point mutations in genes encoding intracellular proteins. Short peptide fragments of these proteins, after intracellular processing and presentation at the cell surface as MHC ligands, can elicit T cell immunoreactivity. Further, the presence of tumor infiltrating lymphocytes (TIL), in particular, CD8^+^ T cells, has been associated with increased survival (Sato et al. 2005; Nelson 2008; Oble et al. 2009; Yamada et al. 2010; Gooden et al. 2011; Hwang et al. 2012), suggesting that the adaptive immune system can mount protective anti-tumor responses in many cancer patients (Kim et al. 2007; Fox et al. 2011). The antigen specificities of tumor-infiltrating T cells remain almost completely undefined (Andersen et al. 2012), but there are numerous examples of cytotoxic T cells recognizing single amino acid coding changes originating from somatic tumor mutations (Lennerz et al. 2005; Matsushita et al. 2012; Heemskerk et al. 2013; Lu et al. 2013; Robbins et al. 2013; van Rooij et al. 2013; Wick et al. 2014). Thus, the notion that tumor mutations are reservoirs of exploitable neo-antigens remains compelling (Heemskerk et al. 2013). For a mutation to be recognized by CD8^+^ T cells, the mutant peptide must be presented by MHC I molecules on the surface of the tumor cell. The ability of a peptide to bind a given MHC I molecule with sufficient affinity for the peptide-MHC complex to be stabilized at the cell surface is the single most limiting step in antigen presentation and T cell activation (Yewdell and Bennink 1999). Recently, several algorithms have been developed that can predict which peptides will bind to given MHC molecules (Nielsen et al. 2003; Bui et al. 2005; Peters and Sette 2005; Vita et al. 2010; Lundegaard et al. 2011), thereby providing guidance into which mutations are immunogenic.

The Cancer Genome Atlas (TCGA) (http://cancergenome.nih.gov/) is an initiative of the National Institutes of Health that has created a comprehensive catalog of somatic tumor mutations identified using deep sequencing. As a member of The Cancer Genome Atlas Research Network, our center has generated extensive tumor RNA-seq data. Here, we have used public TCGA RNA-seq data to explore the T cell immunoreactivity of somatic missense mutations across six tumor sites. This type of analysis is challenged not only by large numbers of mutations unique to individual patients, but also by the complexity of personalized antigen presentation by MHC arising from the extreme *HLA* allelic diversity in the outbred human population. Previous studies have explored the potential immunogenicity of tumor mutations (Segal et al. 2008; Warren and Holt 2010; Khalili et al. 2012), but these have been hampered by small sample size and the inability to specify autologous HLA restriction. Recently, we described a method of HLA calling from RNA-seq data that shows high sensitivity and specificity (Warren et al. 2012). Here, we have obtained matched tumor mutational profiles and *HLA-A* genotypes from TCGA subjects and used these data to predict patient-specific mutational epitope profiles. The evaluation of these data together with RNA-seq-derived markers of T cell infiltration and overall patient survival provides the first comprehensive view of the landscape of potentially immunogenic mutations in solid tumors.

## Results

### Summary of available data

**Figure 1.**
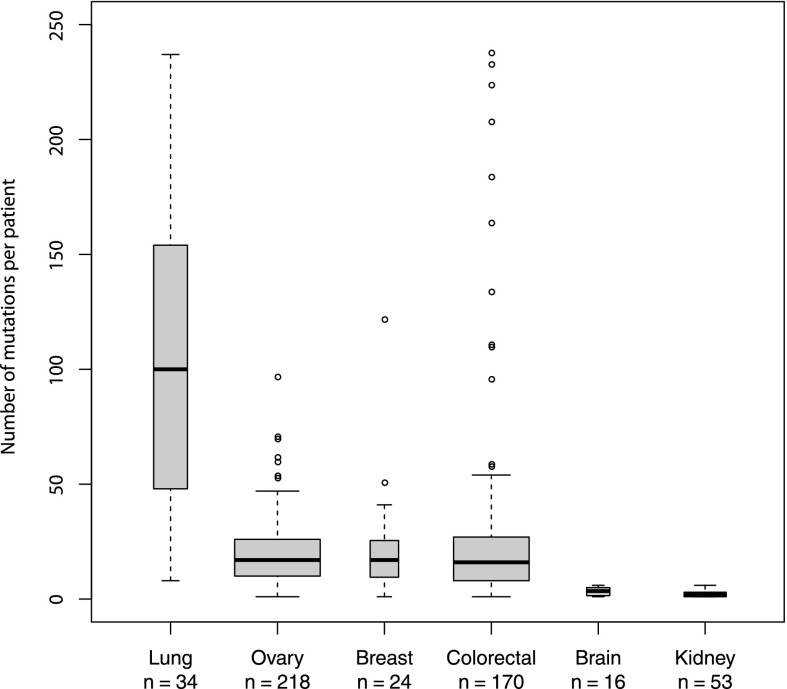
Boxplots showing the number of mutations per patient for each cancer type. The *y*-axis is cut off at 250 mutations for better visualization of the majority of the data. The dark horizontal bar shows the median, whereas the box encompasses the interquartile range (middle 50% of the data). Whiskers reach the farthest data point that is within 1.5× the interquartile range from the nearest box edge (quartile). Box width is proportional to the sample size (lung: 34, ovary: 218, breast: 24, colorectal: 170, brain: 16, kidney: 53).

Raw TCGA RNA-seq data plus clinical metadata and complete profiles of sequence-verified missense mutations were obtained with permission from the Cancer Genomics Hub (https://cghub.ucsc.edu). Our analysis covers six tumor sites, including colon and rectum (combined as colorectal), ovary, breast, brain, kidney, and lung. These were the only tumor sites with complete and nonembargoed data at the time of this study. The RNA-seq data were first processed using HLAminer (Warren et al. 2012) to predict, at four-digit resolution, the two *HLA-A* alleles carried by each subject. Data from 515 patients with unambiguous *HLA-A* calls were processed further. The distribution of missense mutation counts across patients with different tumor types is shown in Figure 1. For each of the 22,758 total missense mutations, we evaluated binding of all possible 8- to 11-mer mutant peptide variants to autologous *HLA-A* encoded MHC proteins using the Immune Epitope Database (IEDB) T Cell Epitope-MHC Binding Prediction Tool (Vita et al. 2010) (http://www.iedb.org/). We focused our analysis on *HLA-A* alleles because (1) MHC I proteins (encoded by *HLA-A, -B*, and -*C* genes) present antigens to CD8^+^ cytotoxic T cells, which are the subset of T cells most strongly linked to patient survival, and (2) *HLA-A* alleles of MHC I yield the most accurate peptide binding affinity predictions by IEDB and most other algorithms due to the abundance of *HLA-A*-specific training data (Hoof et al. 2009). All mutational data, RNA-seq derived *HLA-A* calls, IEDB epitope predictions, RNA-seq-derived gene expression values, and clinical metadata were compiled in a MySQL database for analysis.

### *CD8A* expression is associated with survival

**Figure 2.**
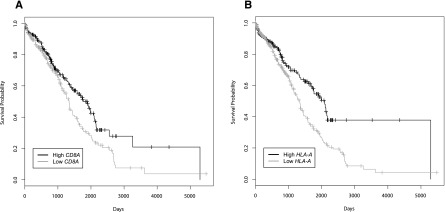
Overall survival for patients based on *CD8A* or *HLA-A* expression. Kaplan-Meier curves were constructed to look at the difference in survival of patients (*n* = 512) with low and high expression levels of *CD8A* (*A*) or *HLA-A* (*B*). Patients were split into two groups based on the median expression value. Patients with high expression showed increased survival compared to those with low expression of either (*A*) *CD8A* (HR = 0.71, 95% CI = 0.53 to 0.94, *P* = 1.7 × 10^−2^) or (*B*) *HLA-A* (HR = 0.59, 95% CI = 0.44 to 0.81, *P* = 8.6 × 10^−4^). Tick marks on the graph denote the last time survival status was known for living patients.

We first asked if we could reproduce the known association between increased numbers of tumor-infiltrating CD8^+^ T cells and increased overall survival (Sato et al. 2005; Nelson 2008; Oble et al. 2009; Yamada et al. 2010; Gooden et al. 2011; Hwang et al. 2012). CD8^+^ TIL levels are usually measured by immunohistological staining. To interrogate RNA-seq data, we used the expression of *CD8A* (one component of the CD8 dimer) as a surrogate for CD8^+^ TIL levels. We observed significantly higher overall survival for patients with high *CD8A* expression than for those patients with low *CD8A* expression (HR = 0.71, 95% CI = 0.53 to 0.94, *P* = 1.7 × 10^−2^) (Fig. 2A). Likewise, the data recapitulated the known association between high *HLA-A* expression and improved overall survival (HR = 0.59, 95% CI = 0.44 to 0.81, *P* = 8.6 × 10^−4^) (Fig. 2B; Concha et al. 1991; Ogino et al. 2006; Kitamura et al. 2007; Han et al. 2008; Bijen et al. 2010). Based on these positive findings with established T cell and MHC markers, we proceeded to evaluate candidate peptide epitopes, which represent the third molecular component required for T cell recognition and destruction of target cells.

### The abundance of tumor missense mutations is not associated with survival

**Figure 3.**
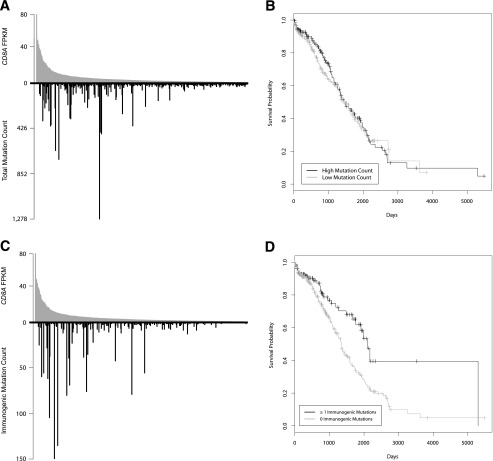
The total number of mutations in tumors is not associated with survival, while the number of predicted immunogenic mutations is associated with survival. (*A*,*C*) A “skew plot” was made for all patients (*n* = 515), ordering patients along the *x*-axis according to their *CD8A* expression. Each patient’s *CD8A* expression was plotted *above* the *x*-axis, and total mutation count (*A*) or predicted immunogenic mutation count (*C*) was plotted *below* the *x*-axis. 73.6% of the total mutation count belonged to patients with above median *CD8A* expression (*P* = 2.0 × 10^−6^), and 84.7% of the total predicted immunogenic mutation count belonged to patients with above median *CD8A* expression (*P* = 1.0 × 10^−6^). (*B*,*D*) Kaplan-Meier curves were constructed to look at the difference in survival between patients with low versus high numbers of mutations. Patients (*n* = 468) were split into two groups based on the median mutation count. There was no difference in survival between the two groups when stratifying on total mutation count (*B*) (HR = 0.91, 95% CI = 0.68 to 1.23, *P* = 5.5 × 10^−1^), but there was a statistically significant difference between the two groups when stratifying on predicted immunogenic mutation count (*D*) (HR = 0.53, 95% CI = 0.36 to 0.80, *P* = 2.1 × 10^−3^). Tick marks on the Kaplan-Meier graphs denote the last time survival status was known for living patients.

Initially, we asked if there is a relationship between overall mutation count and CD8^+^ TIL. Ranking patients by decreasing *CD8A* expression and displaying the mutation count for each patient’s tumor revealed a skewed distribution whereby tumors with low *CD8A* expression had sparse mutations and tumors with high mutation counts were among those with elevated *CD8A* expression (Fig. 3A). Tumors with above median *CD8A* expression contained 73.6% of the total mutations (*P* = 2.0 × 10^−6^ by iterative randomization and resampling as described in Methods). However, there was no association between total mutation count and overall survival (HR = 0.91, 95% CI = 0.68 to 1.23, *P* = 5.5 × 10^−1^) (Fig. 3B).

### Tumor missense mutations that have predicted immunoreactivity are associated with increased survival

We reasoned that missense mutations yielding peptides with poor MHC I binding would be immunologically silent and hence likely to obscure any association between missense mutations, anti-tumor immunoreactivity, and survival. To address this, we repeated the above analysis focusing on those mutations that were most likely to be immunogenic by several criteria, including (1) the expression of the gene in the tumor bearing the mutation was above the median expression level of that same gene in all tumors, (2) *HLA-A* expression in the tumor bearing the mutation was above the median expression of *HLA-A* in all tumors, and (3) the predicted autologous HLA-A binding affinity of the best scoring peptide containing a given mutation had an IC_50_ value of 500 nM or less. This value has been estimated, experimentally, to be the affinity necessary for an epitope to elicit an immune response (Sette et al. 1994). Applying these filters, the predicted immunogenic mutation count was zero in 334 patients. The remaining 181 patients had predicted immunogenic mutation counts ranging from 1 to 147, with a median of 3. The predicted immunogenic mutation count showed a strong relationship with tumor *CD8A* expression, where tumors with higher numbers of such mutations had higher *CD8A* expression (Fig. 3C). Of all predicted immunogenic mutations, 84.7% were in tumors with above median *CD8A* expression (*P* = 1.0 × 10^−6^). We did not see any relationship between predicted immunogenic mutation count and *CD4* expression by tumors (*P* = 6.9 × 10^−1^) (Supplemental Fig. 1), consistent with the fact that we had assessed epitopes presented by MHC class I, which is recognized exclusively by CD8^+^ T cells. Interestingly, patients with tumors containing at least one predicted immunogenic mutation showed markedly increased overall survival compared to those without predicted immunogenic mutations (HR = 0.53, 95% CI = 0.36 to 0.80, *P* = 2.1 × 10^−3^) (Fig. 3D). To further examine this association, we fit a model including all available prognostic factors (age, gender, cancer type, and tumor stage), as well as predicted immunogenic mutations. This model also showed significantly improved overall survival for patients with predicted immunogenic mutations relative to those without (HR = 0.50, 95% CI = 0.31 to 0.80, *P* = 3.9 × 10^−3^), indicating that the effect of predicted immunogenic mutations was independent of the other prognostic factors. Fitting a model which contained an interaction between cancer type and predicted immunogenic mutations did not yield a significant result (*P* = 9.2 × 10^−1^), indicating that the prognostic effect is not limited to a specific cancer diagnosis.

Given that tumor *HLA-A* expression alone is a known indicator of favorable patient survival (Fig. 2B), we asked if the number of predicted immunogenic mutations provides additional predictive value independent of *HLA-A* expression. After removing the *HLA-A* expression requirement from the definition of a predicted immunogenic mutation, we fit a model including all prognostic factors to the subset of patients with high (above median) tumor *HLA-A* expression. Within this subset of patients, we observed that patients with at least one predicted immunogenic mutation had a significantly lower relative risk of death than those without (HR = 0.44, 95% CI = 0.22 to 0.88, *P* = 2.0 × 10^−2^). Evaluating the reciprocal group of patients with low (below median) *HLA-A* expression, where the potential of immunogenic mutations to elicit bona fide anti-tumor responses is expected to be curtailed, there was no significant association between the presence of predicted immunogenic mutations and survival (HR = 1.30, 95% CI = 0.83 to 2.04, *P* = 2.6 × 10^−1^). The results from all survival analyses are summarized in Table 1.

### Predicted immunogenic mutation counts correlate with the expression of T cell exhaustion markers

**Figure 4.**
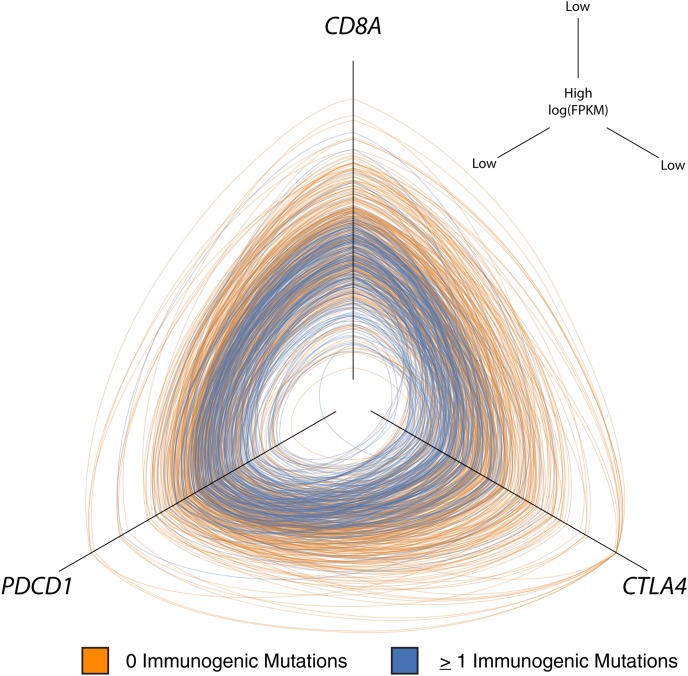
Hive plot showing that tumors with high immunogenic mutation counts have higher expression of *CD8A, PDCD1,* and *CTLA4*. On each axis is the log expression value (log[FPKM]) for *CD8A* (*top*), *PDCD1* (*left*), and *CTLA4* (*right*). Values go from small to large moving toward the *center* of the plot. Each ring represents one patient, and the intersection with the axis represents that patient’s value for that axis. Patients with zero predicted immunogenic mutations are colored orange, and patients with at least one predicted immunogenic mutation are colored blue. Blue rings tend to cluster around the *center* of the plot, indicating concordance between increased predicted immunogenic mutation count and elevated *CD8A, PDCD1,* and *CTLA4* expression (*P* = 1.0 × 10^−6^).

PDCD1 and CTLA4 are T cell surface molecules that can inhibit anti-tumor T cell responses (Schneider et al. 2006; Blank and Mackensen 2007). Blockade of these inhibitory receptors by targeted monoclonal antibodies can disinhibit anti-tumor immunity and improve clinical outcomes (Hodi et al. 2003, 2008, 2010; Hamanishi et al. 2007; Mansh 2011; Brahmer et al. 2012; Topalian et al. 2012; ). Given that many patients in the current study had clinically significant cancer despite having predicted immunogenic mutations and CD8^+^ TIL, we asked if there was an association between immunogenic mutation load and expression of *PDCD1* or *CTLA4*. We found that patients with higher numbers of predicted immunogenic mutations had increased expression of not only *CD8A* but also *PDCD1* and *CTLA4.* Displaying these values in a three-way hive plot (Krzywinski et al. 2012) highlights the association between these T cell markers and immunogenic mutation load (Fig. 4). Significance was assessed by iterative randomization and resampling (as described in Methods). Of all tumors with predicted immunogenic mutations, 45.9% had above median expression of all three of *PDCD1, CTLA4,* and *CD8A* (*P* = 1.0 × 10^−6^).

## Discussion

The adaptive immune system opposes tumor development, and the elicitation of immunogenic cell death is a key component of both targeted immunotherapies and conventional treatment modalities including radiation and chemotherapy (Kroemer et al. 2013). There is a robust association between T cell infiltration of solid tumors and favorable patient outcomes. Missense variants are the most frequent type of oncogenic mutation, which raises the question of whether missense mutations also underlie tumor immunoreactivity. Exome analysis in mice has revealed specific missense mutations that encode MHC class I presented mutational epitopes that are capable of eliciting T cell-mediated tumor rejection (Castle et al. 2012; Matsushita et al. 2012). Moreover, human tumor exome sequencing studies have identified mutational epitopes recognized by autologous CD8^+^ TIL (Heemskerk et al. 2013; Robbins et al. 2013; van Rooij et al. 2013; Wick et al. 2014). However, from these investigations it appears that missense mutations with demonstrable endogenous immunoreactivity are relatively rare. They are a small minority of total missense mutations. It is likely the case that only one or a few mutations per tumor are immunodominant, and tumors with a higher mutational burden simply have an increased likelihood of bearing a highly immunogenic mutation. This is consistent with our results, where total mutations (Fig. 3A) greatly outnumber mutations that are predicted to be immunogenic (Fig. 3C), but the distributions are similar. Looking at cancers individually (Supplemental Fig. 2), it is interesting that colorectal tumors, many of which had very high mutational loads, showed the strongest association between predicted immunogenic mutation counts and *CD8A* expression. Unfortunately, however, in the current meta-analysis the number of subjects varied widely among cancer types. A comprehensive evaluation of immunogenic mutations specific to individual cancer types remains an important topic for future study.

Our meta-analysis focused exclusively on missense mutations because, in addition to these being most abundant, they were sequence-verified and therefore of high confidence. Moreover, they were amenable to evaluation using existing computational epitope prediction tools. We observed that nearly all patient tumors with high missense mutation counts also had elevated CD8^+^ TIL, inferred by *CD8A* expression, and elevated counts of predicted immunogenic mutations. However, the association was directional, with many tumors having high CD8^+^ TIL but few or no predicted immunogenic mutations. This suggests that while the expression of immunogenic missense mutations may induce CD8^+^ TIL responses in some tumors, in other tumors CD8^+^ TIL may be attracted by other classes of mutation or other factors altogether. In patients with hereditary nonpolyposis colorectal cancer, microsatellite instability is the major determinant of dense tumor infiltration by activated CD8^+^ T cells (Dolcetti et al. 1999); thus, a mutator tumor phenotype may, in general, enhance immunoreactivity. Other classes of potentially immunogenic mutations require exploration, such as gene fusions resulting from genomic rearrangements. Instances of tumors with high CD8^+^ TIL but few immunogenic mutations may also be due to immune editing (Matsushita et al. 2012; Vesely and Schreiber 2013). Specifically, tumor cells bearing highly immunogenic mutations may have been selectively eliminated by T cells, resulting in accumulation of CD8^+^ TIL but fewer immunogenic mutations remaining to be detected.

The results of the present study have clinical implications. We have shown that patients with tumors bearing missense mutations predicted to be immunogenic have a survival advantage (Fig. 3D). These tumors also show evidence of higher CD8^+^ TIL, which suggests that a number of these mutations might be immunoreactive. The existence of these mutations is encouraging because, in principle, they could be leveraged by personalized therapeutic vaccination strategies or adoptive transfer protocols to enhance anti-tumor immunoreactivity. Likewise, patients with tumors showing naturally immunogenic mutations and associated TIL are potential candidates for treatment with immune modulators such as CTLA4- or PDCD1-targeted antibodies. There is evidence that such therapies are most effective against tumors infiltrated by T cells (Moschos et al. 2006; Hamid et al. 2009). Our results indicate that tumors bearing predicted immunogenic mutations have not only elevated *CD8A* expression (Fig. 3C) but also elevated expression of *CTLA4* and *PDCD1* (Fig. 4), reinforcing the notion that these patients may be optimal candidates for immune modulation. Importantly, we observed that tumors with low levels of CD8^+^ TIL invariably have far fewer immunogenic mutations. Such patients would be better suited to conventional therapy or to immunotherapies (e.g., chimeric antigen receptor modified T cells) that target nonmutated antigens.

## Methods

### TCGA mutation annotation files

Mutation annotation files (MAF) for unrestricted TCGA cancer sites were downloaded from https://tcga-data.nci.nih.gov/tcgafiles/ftp_auth/distro_ftpusers/anonymous/tumor/. We parsed every available MAF file regardless of level (https://wiki.nci.nih.gov/display/TCGA/Mutation+Annotation+Format+%28MAF%29+Specification); however, only listed variants predicted to yield nonsynonymous missense coding mutations and associated with a predicted RefSeq identifier at the specified genomic location were ultimately tracked. The MAF format specification enabled the selection of putative whole-genome shotgun screen variants that had been verified by orthogonal methods. The screen identified a total of 74,535 verified missense SNVs from 1069 TCGA patients and seven cancer sites, including GBM (glioblastoma multiform) (The Cancer Genome Atlas Research Network 2008), OV (ovarian serous cystadenocarcinoma) (The Cancer Genome Atlas Research Network 2011), LUSC (lung squamous cell carcinoma) (The Cancer Genome Atlas Research Network 2012a), COAD (colon adenocarcinoma) (The Cancer Genome Atlas Research Network 2012b), READ (rectum adenocarcinoma) (The Cancer Genome Atlas Research Network 2012b), BRCA (breast invasive carcinoma) (The Cancer Genome Atlas Research Network 2012c), and KIRC (kidney renal clear cell carcinoma) (The Cancer Genome Atlas Research Network 2013). Parsing scripts, written in PERL, tallied corresponding RNA-seq BAM file names for each of the 1069 TCGA patients for use in conjunction with HLA prediction and gene expression profiling.

### HLA predictions

RNA-seq BAM files for each of the 1069 subjects were downloaded from CGhub and used directly as input for HLAminer (Warren et al. 2012). HLAminer was run with default values, in parallel on a computer cluster. The two highest-scoring four-digit HLA predictions for the *HLA-A* locus were retained (highest score at ranks 1 and 2). Patients with four-digit HLA predictions that were ambiguous, that is, with two or more four-digit HLA alleles scoring equally, were excluded from analysis. RNA-seq read length strongly influences the performance of HLA calling, and ambiguous HLA calls from tumor types where only short reads (50 nt) were available (lung, breast, and kidney) represented the largest source of attrition of TCGA subjects from the meta-analysis. HLAminer predictions, including the genes, rank, group allele, coding allele, score, expect value, confidence, and number of predictions, were stored in a MySQL relational database. A custom script was developed to integrate the automated HLA predictions with SNV-specific information and used as input for HLA epitope predictions.

### HLA ligand binding predictions

A tab-separated file that listed all 74,535 filtered SNVs along with the predicted amino acid coding mutation and protein sequence was split by cancer type and each used as input for PERL scripts designed to query IEDB (http://www.iedb.org/) offline (http://tools.immuneepitope.org/analyze/html_mhcibinding20090901B/download_mhc_I_binding.html) as previously described (Warren and Holt 2010). Briefly, entire protein sequences were submitted in their mutated form and default settings were used for analysis. When supported, 8- to 11-mer peptide predictions were selected, each with a specific HLA allele determined computationally from RNA-seq data for the patient under scrutiny. The output epitope prediction was captured and parsed, and all peptides encompassing the amino acid of interest were tracked, including binding prediction rank and score.

### Gene expression from RNA-seq data

Raw sequence reads were extracted from the 1069 BAM files using bam2fastq v.1.1.0. Extracted reads were subsequently aligned to the human reference genome and transcriptome (hg19, Ensembl v70) using the ultrafast aligner STAR v. 2.3.0e (Dobin et al. 2013) with the following parameters: minimum/maximum intron size set to 30 and 500,000, respectively, noncanonical, unannotated junctions were removed, maximum tolerated mismatches was set to 10, and the outSAMstrandField intronMotif option was enabled. The Cuffdiff command included with Cufflinks v. 2.0.2 (Trapnell et al. 2010) was used to calculate the fragments per kilobase of exon per million fragments mapped (FPKM) (Trapnell et al. 2010) with upper quartile normalization, fragment bias correction, and multiread correction enabled. All other options were set to default.

### Clinical data sets

TCGA clinical data sets were downloaded from https://tcga-data.nci.nih.gov/tcgafiles/ftp_auth/distro_ftpusers/anonymous/tumor/DISEASE_CODE/bcr/biotab/clin/. For each cancer site, we obtained clinical_follow_up_vX.X_XXX.txt and clinical_patient_XXX.txt. The files were parsed and pertinent clinical information extracted and saved into a MySQL relational database.

### Data analysis

Pertinent data was extracted from the MySQL database using custom queries, and the results were saved to tab delimited text files. These files were read into R v. 3.0.1 (R Development Core Team 2013) for further statistical analysis. Colon and rectum cancers were combined for all analyses as colorectal cancer. A single colorectal patient with total mutation count 20.3 standard deviations away from the mean mutation count of all patients was removed from all analysis.

To count the overall number of putatively immunogenic mutations for each patient, we first summed the total number of point mutations which contained a peptide predicted to be presented by the MHC molecules encoded by the *HLA-A* alleles identified, unambiguously, for that patient. The requirement of unambiguous *HLA-A* prediction resulted in a sample size of 515. We then took the “best” peptides for each point mutation, which were those with the highest predicted binding affinity (lowest IC_50_) to its respective autologous MHC variant. We filtered these peptides by keeping those which had an IC_50_ value below 500 nM. We then filtered these peptides to those which were expressed at a level higher than the median expression for their given gene. We further filtered these peptides to those where the *HLA-A* gene expression was higher than the median of all *HLA-A* gene expression values. These cut-offs were selected to maximize the probability that a given peptide was able to be seen by a T cell receptor, in which case it should be highly expressed and bind to an MHC variant that is also highly expressed. The number of peptides which passed these criteria was used as the number of predicted immunogenic mutations for each patient.

### Statistical analysis

We modified a random reassignment method, described previously (Warren et al. 2013), to test the significance of associations with TIL gene expression markers. First, the percent of mutations that belonged to tumors with above median *CD8A* expression was calculated. Next, counts of mutations were randomly reassigned to tumors 1,000,000 times using the boot package (Canty and Ripley 2012) in R. The percent of total mutations belonging to tumors with above median *CD8A* expression was calculated after each random reassignment, and the bootstrap *P*-value was equal to the proportion of randomizations where the number of mutations belonging to tumors with above median *CD8A* expression was equal to or greater than the number of mutations belonging to tumors with above median *CD8A* expression in the original, nonrandomized data. This same method was used to test the significance of associations between the presence of predicted immunogenic mutations and elevated expression of all three genes, *PDCD1, CTLA4*, and *CD8A*.

Survival times were calculated as the number of days from initial pathological diagnosis to death, or the number of days from initial pathological diagnosis to the last time the patient was known to be alive. These times were used in the construction of the Kaplan-Meier survival curves and Cox proportional hazard models. Potential confounders—age, gender, cancer, and tumor stage—were examined. The R survival package (Therneau 2013) was used to construct Kaplan-Meier curves and fit the univariate and multivariate Cox proportional hazard models. Five hundred and twelve patients were used in the survival analysis investigating *CD8A* and *HLA-A* after removing three patients without survival information. The 16 brain tumor patients were excluded from the analysis as they were missing tumor stage information. The 24 breast patients were also excluded from analysis as the low mortality rate (1/24) was not informative. Additionally, seven patients were not used in the survival analysis as their prognostic information was incomplete. This resulted in a sample size of 468 for the multivariate survival analysis.

### Hive plots

An R script was designed to create hive plot input files from the original data, converting from a table format to the graph format, DOT. These input files were imported into jhive v0.0.18 (http://hiveplot.com/distro/jhive-0.0.18.zip) to create the hive plots (Krzywinski et al. 2012).

**Table 1.**
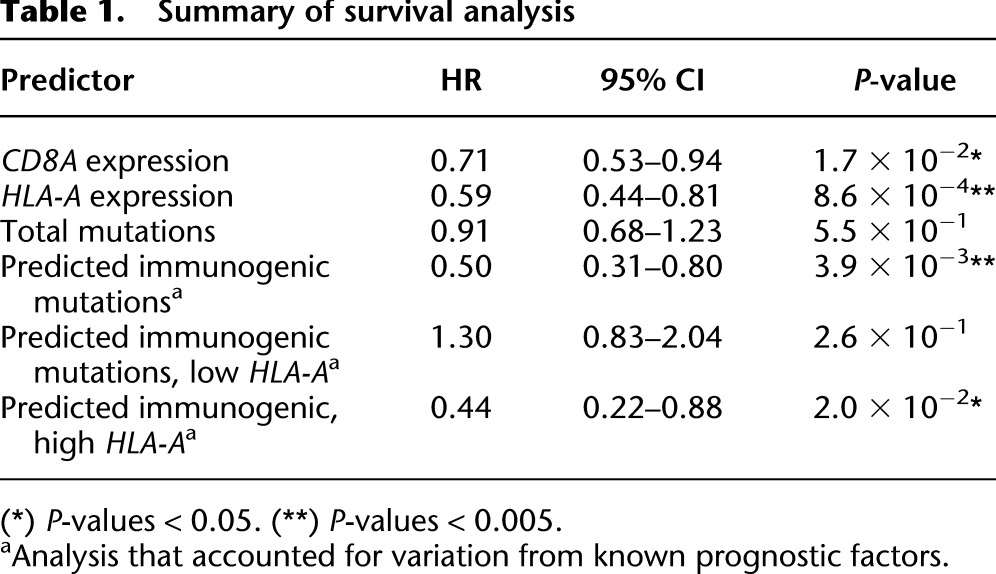
Summary of survival analysis
